# Burden of acute otitis media in primary care pediatrics in Italy: a secondary data analysis from the Pedianet database

**DOI:** 10.1186/1471-2431-12-185

**Published:** 2012-11-29

**Authors:** Paola Marchisio, Luigi Cantarutti, Miriam Sturkenboom, Silvia Girotto, Gino Picelli, Daniele Dona, Antonio Scamarcia, Marco Villa, Carlo Giaquinto

**Affiliations:** 1 Department of Pathophysiology and Transplantation, University of Milan, and Fondazione IRCCS Cà Granda Ospedale Maggiore Policlinico, Milan, Italy; 2 Family Pediatrician Pedianet Project, Padova, Italy; 3 Department of Medical Informatics, Erasmus University Medical Center, Rotterdam, The Netherlands; 4 International Pharmacoepidemiology and Pharmacoeconomics Research Center, Desio, Italy; 5 Department of Pediatrics, University of Padua and Pedianet Project, Padua, Italy; 6 Società Servizi Telematici, Padua, Italy; 7 Local Health Authority ASL Cremona, Cremona, Italy

**Keywords:** Acute otitis media, Incidence, Primary care pediatrics

## Abstract

**Background:**

The incidence of acute otitis media (AOM) vary from country to country. Geographical variations together with differences in study designs, reporting and settings play a role. We assessed the incidence of AOM in Italian children seen by primary care paediatricians (PCPs), and described the methods used to diagnose the disease.

**Methods:**

This secondary data analysis from the Pedianet database considered children aged 0 – 6 years between 01/2003 and 12/2007. The AOM episodes were identified and validated by means of patient diaries. Incidence rates/100 person-years (PY) were calculated for total AOM and for single or recurrent AOM.

**Results:**

The 92,373 children (52.1% males) were followed up for a total of 227,361 PY: 23,039 (24.9%) presented 38,241 episodes of AOM (94.6% single episodes and 5.4% recurrent episodes). The total incidence rate of AOM in the 5-year period was 16.8 episodes per 100 PY (95% CI: 16.7-16.9), including single AOM (15.9 episodes per 100 PY; 95% CI: 15.7-16.1) and recurrent AOM (0.9 episodes per 100 PY; 95% CI: 0.9-0.9). There was a slight and continuously negative trend decrease over time (annual percent change −4.6%; 95%CI: -5.3, -3.9%). The AOM incidence rate varied with age, peaking in children aged 3 to 4 years (22.2 episodes per 100 PY; 95% CI 21.8-22.7). The vast majority of the AOM episodes (36,842/38,241, 96.3%) were diagnosed using a static otoscope; a pneumatic otoscope was used in only 3.7%.

**Conclusions:**

Our data fill a gap in our knowledge of the incidence of AOM in Italy, and indicate that AOM represents a considerable burden for the Italian PCP system. Educational programmes concerning the diagnosis of AOM are needed, as are further studies to monitor the incidence in relation to the introduction of wider pneumococcal conjugate vaccines.

## Background

Acute otitis media (AOM) is one of the most common infectious diseases affecting infants and young children, and one of the main reasons for antibiotic treatment [1-4]. Its diagnosis is often challenging in everyday practice because the symptoms are non-specific or absent, and frequently little attention is paid to the issued guidelines [5-7]. It is estimated that about two-thirds of children aged less than three years have had at least one episode of AOM, and about one-third have had recurrences [8]. The impact on the pediatric health care system is considerable at both societal and family level [9].

The incidence of AOM varies from country to country both because of geographical variations and of different study designs, reporting and settings [10-15]. Annual OM rates are still remarkably high in the USA, although there has been a decline over the last decade since the introduction of heptavalent pneumococcal vaccine [16]. The European data indicate a somewhat lower incidence but there are not very many published studies, and there very few data relating to Italian children [17-20].

In the last decade the introduction of pneumococcal conjugate vaccines has dramatically reduced invasive pneumococcal diseases [21]. In light of the potential impact also against non-invasive diseases such as middle ear infections, there has been a continuing interest in defining the incidence and burden of AOM in different populations, including those in which the offer and coverage of heptavalent pneumococcal conjugate vaccine has been considerably heterogeneous [22-25].

The aims of this study were to assess the incidence of AOM in Italian children seen by primary care pediatricians (PCPs) and to describe the methods used to diagnose the disease.

## Methods

### Study design and setting

This secondary data analysis was based on data coming from the Pedianet database (http://www.pedianet.it), a pediatric general practice research database that contains the clinical, demographic, prescription and outcome data of the children routinely seen by about 130 PCPs equally distributed throughout Italy (all Italian children aged less than six years are registered with a PCP as part of the country’s national health service), and which has been used for various epidemiological and pharmacovigilance studies [26-29]. The data are generated during routine patient care using common software (JuniorBit ®), and are anonymously sent monthly to a centralised database for validation. The reasons for the contacts and diagnoses (free text or coded using the ICD-9 system) are recorded in the medical file, and the database also contains information about specialist referrals, procedures, hospitalisations, medical examinations, health status (according to the Guidelines of Health Supervision of the American Academy of Pediatrics), and centile diagrams. As of December 2008, the Pedianet database stored data relating to 250,000 children, including the findings of about 3,000,000 examinations, 2,600,000 diagnoses, 1,800,000 drug prescriptions and 30,000 hospitalisations. The study and the access to the database were approved by the Internal Scientific Committee.

### Study population

All children aged 0–6 years who were registered with one of the 108 out of 130 Pedianet PCPs who agreed to participate in the study between January 1^st^, 2003 to December 31, 2007 were included. Any children whose parents did not actively provide their informed consent were excluded from the study.

### Case definition and identification

The cases of AOM were initially identified from coded diagnoses (ICD-9: 380 to 389). In order to identify any cases not reported in the diagnosis fields, we searched the files describing hospitalisations, diagnoses, diaries, specialist examinations and reasons for examinations using a sensitive text string that included the words “acute otitis”, “AOM”, “myringitis”, “ear pain”, “fever”, “ear discharge”, “rhinitis”, and “vomiting”. Once identified, these cases were recoded using the ICD9 classification. All of the potential episodes were manually evaluated and validated in order to exclude any false positive cases from the free text searches. The visits were considered to be associated with a single AOM episode if they occurred within one month of the initial diagnosis. One patient could have had more than one episode of AOM if they were more than 30 days apart. Recurrent otitis media (rAOM) was defined as a third episode of AOM occurring within a period of six months or a fourth episode occurring in a period of 12 months, regardless of the age of the child.

The following information was sought in the clinical record: the presence of any symptoms at the time of the first examination and the diagnostic procedures used (including static otoscopy, pneumatic otoscopy, tympanometry, blood sampling for WBC counts and the rapid determination of C-reactive protein level).

The cases of AOM were classified on the basis of diagnostic accuracy:

a) Level 1: diagnosed by a physician and documented as such in the medical file or another source document without any other specification;

b) Level 2: level 1 definition plus a report of the visual appearance of the tympanic membrane (i.e. redness, bulging, loss of light reflex), the presence of acute middle-ear effusion (as shown by otoscopy or tympanometry), and the presence of at least two of the following signs or symptoms: ear pain, ear discharge, cough, rhinitis, hearing loss, lethargy, irritability, anorexia, vomiting, diarrhea, fever (axillary temperature ≥38.0°C, rectal temperature ≥38,5°C) or analgesic/antipyretic therapy for previous fever;

All of the children were followed from the date of registration with the PCP, the date of birth, or the start of the study period (whichever was the latest) until death, moving away, or the age of six years, whichever was the earliest.

### Statistical analysis

The person-time of follow-up in the source population was calculated by age group (0–1, 1–2, 2–3, 3–4, 4–5, 5–6 years) and calendar year, and formed the denominator for calculating incidence. The cases (as defined above) provided the numerator. The incidence of AOM was calculated by dividing the number of cases by the accrued person-time (patient-years, PY). Age, gender and season-specific incidence rates were also calculated. The 95% confidence intervals around the rates were based on Poisson’s distribution. In order to characterise the trend of the incidence rates over time, the annual percent change (APC) was estimated using a Poisson model with trigonometric factors accounting for seasonality.

## Results

### Source population

The study population consisted of 92,373 children (52.1% males) aged 0–6 years who were followed up for a total of 227,361 PY: 23,039 children (24.9%) presented 38,241 primary diagnoses of AOM (36,180 single episodes [94.6%] and 2061 recurrent episodes [5.4%]).

The vast majority of AOM episodes (36,842/38,241; 96.3%) were classified as level 2 diagnostic accuracy, which was strengthened by the fact that 34,138 (94.3%) of the single episodes and 1892 (91.8%) of the recurrent episodes was accompanied by the report of a sign or symptom. The rest of the episodes were classified as having level 1 diagnostic accuracy.

### Incidence of AOM

**Figure 1:**
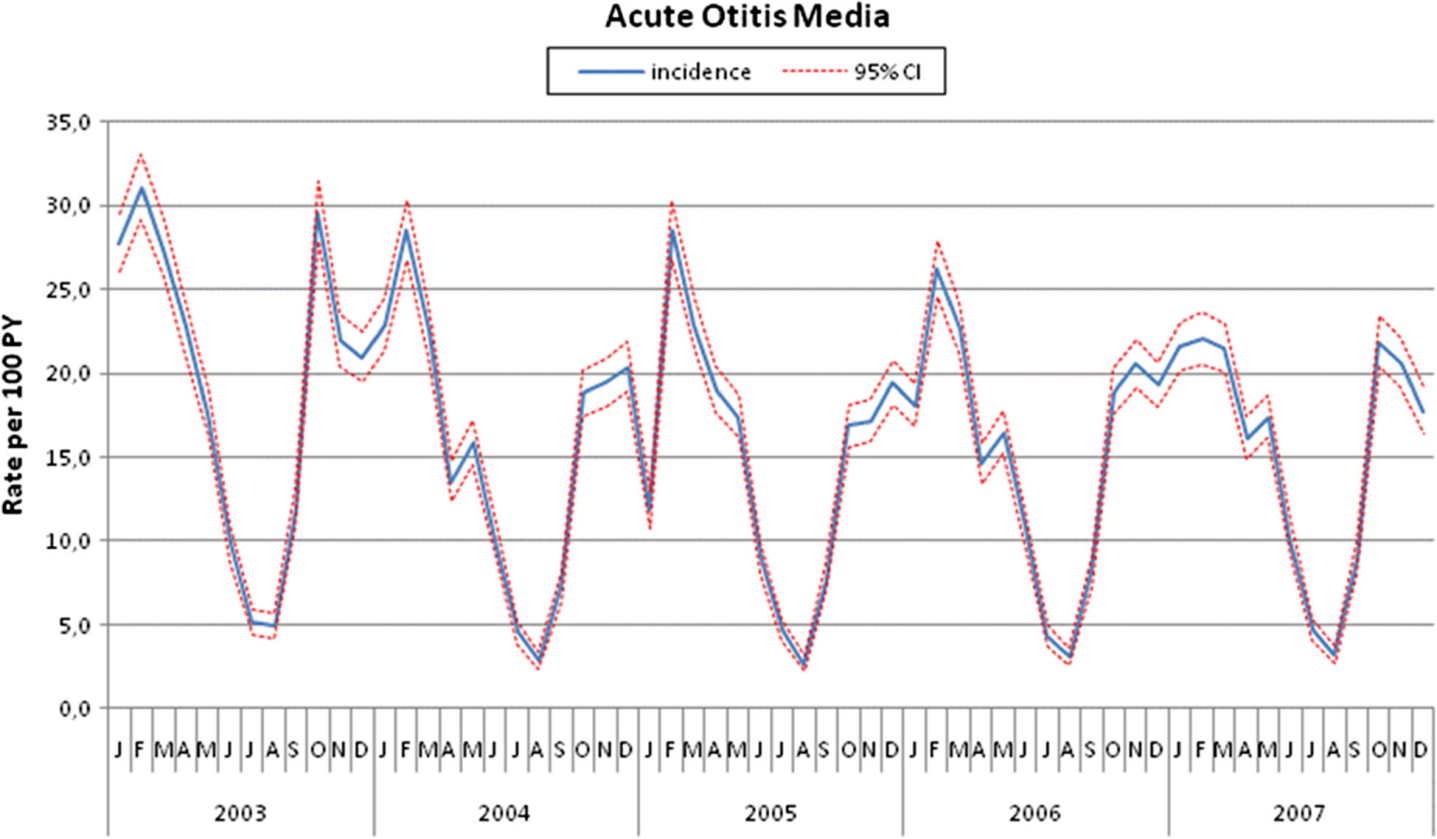
Incidence (per 100 PY) of AOM by month and year.

Table 1 shows the incidence of AOM episodes by type of AOM. The total rate in the 5-year period was 16.8 episodes per 100 PY, including single episodes (15.9 per 100 PY) and recurrent episodes (0.9 per 100 PY). The overall incidence was slightly higher in males (16.5 episodes per 100 PY; 95% CI: 16.3-16.7) than in females (15.3 episodes per 100 PY; 95% CI: 15.0-15.5; p <0.001), but there was no gender-related difference in terms of recurrent AOM (0.9 vs 0.9 episodes per 100 PY). The differences in incidence by gender did not change substantially over the years. The incidence was highest in the winter, but also high in the autumn and spring (Figure 1). The long-term rates showed a negative trend (APC −4.6%; 95%CI: from −5.3 to −3.9%). Although most of the decrease occurred in the first two years, Joinpoint regression did not reveal any significant change in the trend.

**Table 1:** Incidence rates of acute otitis media by year and type of episode

	**2003**	**2004**	**2005**	**2006**	**2007**	**Total**
No. of children followed up	49782	53152	58426	57092	55454	92373
Person time (years)	41251.3	43459.5	48314.8	47956.7	46378.7	227361.0
**Total AOM**						
No. of children	6614	5928	6243	6490	6346	23039
No. of episodes	8138	7173	7496	7815	7619	38241
Incidence rate*	19.7 (19.3-20.2)	16.5 (16.1-16.9)	15.5 (15.2-15.9)	16.3 (15.9-16.7)	16.4 (16.1-16.8)	16.8 (16.7-16.9)
**Single AOM**						
No. of children	6438	5648	5951	6142	6023	21866
No. of episodes	7910	6729	7084	7293	7164	36180
Incidence rate*	19.2 (18.8-19.6)	15.5 (15.1-15.9)	14.7 (14.3-15.0)	15.2 (14.9-15.6)	15.4 (15.1-15.8)	15.9 (15.7-16.1)
**Recurrent AOM**						
No. of children	176	280	292	348	323	1173
No. of episodes	228	444	412	522	455	2061
Incidence rate*	0.6 (0.5-0.6)	1.0 (0.9-1.1)	0.9 (0.8-0.9)	1.1 (1.0-1.2)	1.0 (0.9-1.1)	0.9 (0.9-0.9)

* Incidence rate (per 100 PY, 95% CI).

**Figure 2:**
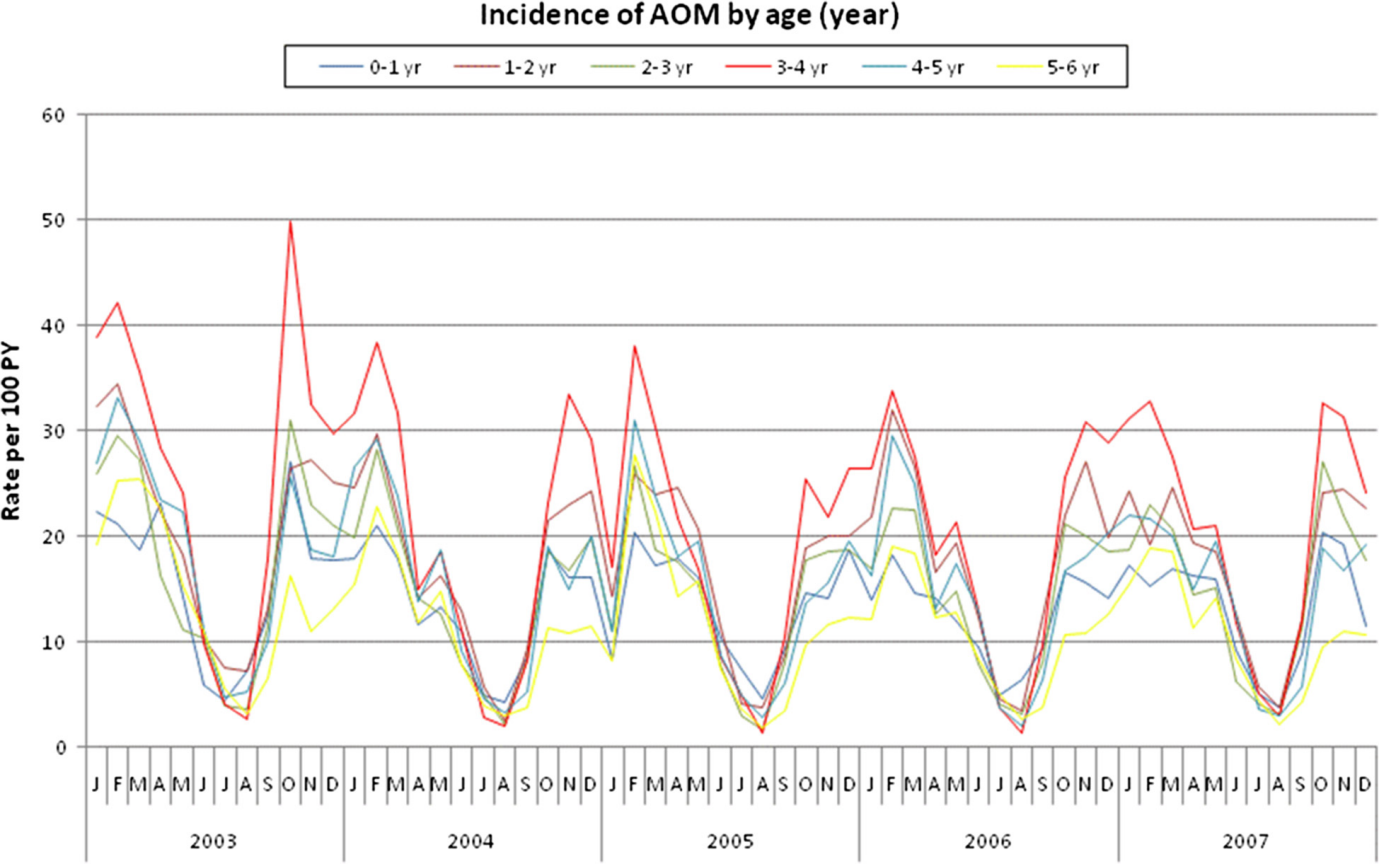
Incidence (per 100 PY) of AOM by age (year) and calendar year.

Table 2 shows the incidence of AOM per 100 PY by age group. The age group showing the highest rates of single and recurrent AOM was 3–4 years, followed by 1–2 years, whereas the lowest rate was found in children aged 5–6 years (Table 2). Seasonal variations were observed at all ages (Figure 2).

**Table 2:** Length of follow-up (person-time) and incidence rates by type of acute otitis media and age group

	**0-1 year**	**1-2 years**	**2-3 years**	**3-4 years**	**4-5 years**	**5-6 years**	**Total**
No. of children followed up	69289	40106	41332	41683	41575	39921	92373
Person time (years)	33241.9	37248.0	38950.5	39798.1	40151.6	37970.9	227361.0
**Total AOM**							
No. of children	3807	5673	5107	6991	5448	3935	21947
No. of episodes	4709	7212	6180	8848	6664	4628	38241
Incidence rate*	14.2 (13.8-14.6)	19.4 (18.9-19.8)	15.9 (15.5-16.3)	22.2 (21.8-22.7)	16.6 (16.2-17.0)	12.2 (11.8-12.5)	16.8 (16.7-17.0)
**Single AOM**							
No. of children	3796	5576	5024	6915	5330	3855	21866
No. of episodes	4550	6703	5858	8376	6285	4408	36180
Incidence rate*	13.7 (13.3-14.1)	18.0 (17.6-18.4)	15.0 (14.7-15.4)	21.0 (20.6-21.5)	15.7 (15.3-16.0)	11.6 (11.3-12.0)	15.9 (15.7-16.1)
**Recurrent AOM**							
No. of children	118	333	216	347	272	171	1173
No. of episodes	159	509	322	472	379	220	2061
Incidence rate*	0.5 (0.4-0.6)	1.4 (1.3-1.5)	0.8 (0.7-0.9)	1.2 (1.1-1.3)	0.9 (0.9-1.0)	0.6 (0.5-0.7)	0.9 (0.9-0.9)

* Incidence rate (per 100 PY, 95% CI).

### Characteristics of AOM episodes

Table 3 shows the occurrence of symptoms by type of AOM and age group. Ear pain was reported in 55.6% of the children with single AOM and 50.4% of those with recurrent AOM, and fever (≥38 °C) in respectively 49.9% and 39.4%. Overall, the proportion of children with ear pain increased with age, while the proportion of children with fever decreased with age in both groups. Cough was the third most commonly encountered symptom in the children with single or recurrent AOM (26.9% *vs* 33.3%; p = <0.0001), being highest in younger children. Spontaneous otorrhea was reported in about 6% of the children in both groups.

**Table 3:** Occurrence of symptoms (%) in children with single and recurrent acute otitis media by age group

	**0 – 1 year**		**1 - 2 years**		**2-3 years**		**3-4 years**		**4-5 years**		**5-6 years**		**Total**	
	AOM*	**rAOM°**	**AOM**	**rAOM**	**AOM**	**rAOM**	**AOM**	**rAOM**	**AOM**	**rAOM**	**AOM**	**rAOM**	**AOM**	**rAOM**
**Total number of episodes**	4550	159	6703	509	5858	322	8376	472	6285	379	4408	220	36180	2061
**Total number with ≥1 symptom**	3650 (80.2)	132 (83.0)	6424 (95.8)	446 (87.6)	5635 (96.2)	262 (81.3)	8338 (99.5)	435 (92.2)	6048 (96.2)	355 (93.7)	4043 (91.7)	173(78.6)	34138 (94.3)	1892 (91.8)
**Ear pain**	15.7	14.3	26.7	24.1	57.4	58.5	68.7	57.0	76.3	74.8	79.6	66.9	55.6	50.4
**Fever ≥38 °C**	60.3	54.9	63.4	50.6	52.2	37.1	46.4	37.1	40.5	26.8	36.0	33.9	49.9	39.4
**Cough**	36.9	49.5	35.0	39.8	27.0	35.4	24.5	33.9	20.9	24.4	18.4	17.3	26.9	33.3
**Rhinitis**	16.2	14.3	15.7	11.1	10.6	11.4	8.5	7.3	8.0	5.7	7.2	5.5	10.8	9.0
**Otorrhea**	6.8	5.5	6.7	6.5	5.9	6.6	4.8	5.6	4.7	6.5	4.7	3.9	5.5	6.0
**Vomiting**	2.9	3.3	3.2	2.2	2.3	0.9	2.3	2.1	2.4	0.8	2.1	0.8	2.5	1.6

*AOM: single acute otitis media; ° rAOM; recurrent acute otitis media.

### Diagnostic methods

Most of the cases (96.3%) were diagnosed using a static otoscope; pneumatic otoscopy was only used in 3.7% of the single episodes and 1.9% of the recurrent episodes. Tympanometry was used as an adjunctive diagnostic method in 1.2% of the single episodes and in 2.5% of the recurrent episodes. Blood samples were drawn in a minority of children for WBC counts (0.3% of the single episodes and 0.1% of the recurrent episodes) and C-reactive protein levels (0.3% of the single episodes and 0.3% of the recurrent episodes).

## Discussion

This is the first large-scale study describing the incidence of AOM in Italian children diagnosed by PCPs. The incidence slowly and slightly decreased between 2003 and 2007, with peaks fluctuating around 16%. The seasonal incidence paralleled the seasonal variations in upper respiratory tract infections. The age group showing the highest rates of single and recurrent episodes was 3–4 years, whereas the one with the lowest rate was 5–6 years.

### Strengths and limitations

The main strength of this study is the use of data covering a five-year period taken from a validated research database of a large national network of PCPs, who have been used to collaborating in order to produce epidemiological data for the most common diseases over the last ten years [26-29]. One of the main strengths of Pedianet is the reliability of its baseline population of children registered with individual PCPs working within the Italian national health service. This allowed the incidence of AOM to be calculated on the basis of person-time by age groups, and provides a precise picture of the disease in the field. Furthermore, as it was a secondary data analysis, no diagnostic criteria were imposed and the cases were identified on the basis of real-world individual PCP criteria, and therefore reflects the real diagnostic and management approach of Italian PCPs to AOM. An additional strength is the fact that all of the episodes of AOM were reviewed and validated: the cases were not just based on coded diagnoses, but traced by means of a manual search in the clinical record. Finally, the study period preceded the introduction of the Italian AOM guidelines [30], thus avoiding another possible confounding factor capable of having an impact on PCP behaviour.

The study has a few limitations, including its retrospective nature and the fact that the PCPs may not have used the same definition of AOM. Some epidemiological variables associated with an increased risk of AOM, such as exposure to passive smoking, number of siblings, day care attendance or prolonged use of a pacifier [31,32], were not recorded but this was beyond the scope of the study.

There was also no information concerning the pneumococcal or influenza vaccination status of the children. Heptavalent pneumococcal conjugate vaccine was gradually introduced into Italy in late 2005 [33], with different regions adopting different policies that varied from the vaccination of specific groups to the vaccination of all infants. However, about half of the PCPs participating in the study were working in regions in which it was initially recommended only for specific high-risk groups, and it is unlikely that the lack of the information regarding vaccination would have been a major confounding factor in such a setting.

We did not record episodes of AOM not seen by PCPs but diagnosed in an Emergency Room (ER). However, as PCPs are free of charge in Italy, it is likely that only a negligible proportion of children would have only been seen in an ER.

Finally, we did not study the severity of the AOM episodes because, although important, there is no consensus concerning the best clinical severity score [29].

### Comparison with other studies

The incidence of AOM is lower than that reported in Northern Europe [34-36] and the USA [37,38], but similar to the rates observed in The Netherlands [19] and the Czech Republic [22]. The prospective cohort EPI AOM study found similar differences between the incidence of AOM in Italy and four other European countries in 2008 [20]: the incidence of AOM in children aged less than five years was 19.5% (95% CI 17.1-22.2%) in Italy, which is not remarkably higher than that described here (16.8%). This finding, which could have been expected given the retrospective design of our study, is quite reassuring about the validity of our data.

There are several possible explanations for the lower incidence rates observed in our population. We only considered validated episodes and not visits; furthermore, multiple examinations within 30 days of the first reported diagnosis were considered as being related to just one episode (although these were reported very rarely). The better climate of Italy in comparison with Northern Europe may also be a factor, but this does not apply to Spain. The functioning of the Italian NHS, which provides specialised primary care to all children free of charge and is funded by paying PCPs on the basis of the number of children registered with them, regardless of the number of accesses, avoids any possible risk that they increased the number of visits to increase their income or that parents avoided seeking care for economic reasons. We cannot exclude the possibility that at least a few AOM cases were seen in an ER without being first reported by a PCP, but these would very likely have been identified because a follow-up examination by a PCP is very often recommended after ER discharge.

The lower incidence of AOM in Italy cannot be attributed to parents not consulting a physician as a result of the 2004 introduction of the US guidelines on AOM [2], which suggested observation without the use of antibiotics, because it has been demonstrated that Italian pediatricians do not generally follow these recommendations [7]. In addition, racial factors, known to influence the incidence of severe and chronic suppurative otitis media in selected racial groups such as Australian aboriginal children and Native Americans, cannot be an explanation because they have no role in the incidence of AOM in white children [39,40]. Finally, it is unlikely that there was an impact of influenza vaccine, known to be able to reduce the incidence of AOM [41], because influenza vaccine is not routinely recommended for Italian children and only a few are actually vaccinated [42].

Pediatric OM consultations have decreased in Europe and the US over the last 10 years [11,16,43-45]. The slight but continuous decrease over time in our population is very similar to that observed in other surveillance studies. One possible confounder may have been the introduction of heptavalent pneumococcal vaccine in late 2005: however, any change before then cannot be attributed to the vaccine and, in the post-introduction period, there was no obviously accelerated decrease in the incidence of AOM. It can be speculated that the low heptavalent pneumococcal vaccine coverage in the immediate post-introduction would not have had a substantial impact on AOM in the population as a whole.

The peak incidence of AOM was in children aged 3–4 years. This conflicts with published reports indicating that children aged up to two years are most frequently affected by AOM. It is likely that the risk arising from exposure to other children was less in our survey because day care attendance in Italy typically begins at three years of age and the small size of the average Italian family (2.47 members) [46] reduces the risk of exposure to other children in the youngest age groups.

Diagnosis is a challenge for PCPs. Half of the children in our study did not present ear pain or fever. This finding (which may be partially due to under-reporting in the database) is in line with those of previous studies [47,48] and supports the need for diagnostic otoscopic skills to avoid the risk of underestimating true AOM. On the other hand, cough and rhinitis were present in more than one-third of the children, thus supporting the belief that OM begins as a viral infection of the nasopharynx and the Eustachian tube and is therefore part of a more wide-ranging respiratory disease [49]. Otorrhea affected a minority of the children: however, this finding is important because it is believed that children with a spontaneously perforated tympanic membrane are more likely to have a poor outcome [50]. Finally, vomiting was present in 2% of cases: this prevalence is lower than that observed in other studies, but the difference can be explained by the fact that we required actual gastrointestinal vomiting and not mucous vomiting [48].

Only a minority of the PCPs reported using a pneumatic otoscope, which is considered the primary diagnostic method by most AOM guidelines. This is consistent with the finding of our previous survey concerning the use of diagnostic instruments by Italian physicians [7]. Most Italian PCPs refer the most severe or recurrent cases of AOM (and all cases of OM with effusion) to an otolaryngologist, and it is therefore likely that they do not feel the need for more accurate diagnostic instruments.

We found it surprising that WBC counts and rapid C-reactive protein testing were included in the diagnostic work-up of children with AOM as it has been demonstrated that they are not useful in differentiating viral from bacterial episodes [51]. However, it is possible that, in these limited cases, the hematological evaluation was made because of the concomitant presence of a secondary infection.

## Conclusions

In brief, our data (which are confirmed by the results of a smaller prospective study) fill a gap in our knowledge concerning the incidence of AOM in Italy and indicate that it represents a considerable burden on the Italian PCP system. Educational programmes concerning the diagnosis of AOM are needed, as are further studies to monitor incidence in relation to the introduction of wider pneumococcal conjugate vaccines.

## Competing interests

No author has conflict of interests to declare. GSK-BIO supported the survey by means of an unrestricted educational grant.

## Authors’ contributions

PM has been involved in analysis and interpretation of data and in drafting and revising the manuscript. LC, SG, GP, DD, AS, have made substantial contributions to conception and design, and acquisition of data, and analysis and interpretation of the data. MS has made substantial contributions to conception and design and has been involved in revising critically the manuscript. MV has been involved in the analysis of data and in drafting the manuscript. CG has made substantial contributions to conception and design, and acquisition of data, and analysis and interpretation of the data and has been deeply involved in revising the manuscript. All authors read and approved the final manuscript.

## Pre-publication history

The pre-publication history for this paper can be accessed here:

http://www.biomedcentral.com/1471-2431/12/185/prepub
